# Evaluating the antioxidant, anti‐inflammatory, and neuroprotective potential of fruiting body and mycelium extracts from edible yellow morel (*Morchella esculenta* L. Pers.)

**DOI:** 10.1111/1750-3841.17619

**Published:** 2025-01-26

**Authors:** Rida Haider, Luisa Agnello, Shahid Masood Shah, Muhammad Sufyan, Nimra Khan, Abdul Nazir, Marcello Ciaccio, Sidra Rehman

**Affiliations:** ^1^ Department of Biotechnology COMSATS University Islamabad (CUI), Abbottabad Campus Abbottabad Pakistan; ^2^ Institute of Clinical Biochemistry, Clinical Molecular Medicine, and Clinical Laboratory Medicine, Department of Biomedicine, Neurosciences and Advanced Diagnostics (BIND) University of Palermo Palermo Italy; ^3^ Department of Bioinformatics and Biotechnology Government College University Faisalabad Pakistan; ^4^ Department of Environmental Sciences COMSATS University Islamabad (CUI), Abbottabad Campus Abbottabad Pakistan; ^5^ Department of Laboratory Medicine University Hospital “P. Giaccone” Palermo Italy; ^6^ Department of Biosciences COMSATS University Islamabad (CUI) Park Road Islamabad Pakistan

**Keywords:** acetylcholinesterase, Alzheimer's disease, molecular docking, *Morchella esculenta*

## Abstract

**Scope:**

This study aimed to assess the antioxidant, anti‐inflammatory, and acetylcholinesterase activities of fruiting bodies (FB) and mycelium (M) extracts of *Morchella esculenta L*. collected from various regions of Pakistan. The samples included Skardu fruiting body (SKFB) and mycelia Skardu (SKM), Malam Jaba fruiting body (MJFB) and Malam Jaba mycelia (MJM), Krair Mansehra fruiting body (KMFB) and Krair Mansehra mycelia (KMM), and Thandiani fruiting body (TFB) and Thandiani mycelia (TM).

**Methods and results:**

The IC_50_ values for free radical scavenging activity of all samples revealed that fruiting body SKFB and MJFB of *M. esculenta* are significantly involved in relieving oxidative stress. Bovine serum albumin conformation destruction assay showed a significantly increased anti‐inflammatory activity of SKFB with an IC_50_ value of 10.94 ± 0.098 µg/mL. The human red blood cell protection assay showed that TFB has a lower EC_50_ value as compared to other samples. KMFB and KMM extracts of *M. esculenta* showed significantly higher anti‐acetylcholinesterase activity compared to the standard drug, donepezil. The lower IC_50_ value of *M. esculenta* extracts suggested higher efficacy for acetylcholinesterase (AChE) inhibition. Enzyme kinetics results showed that KMFB of *M. esculenta* is a competitive inhibitor, while KMM and Donepezil are noncompetitive AChE inhibitors. Further, molecular docking, physicochemical properties and ADMET analysis of *M. esculenta* constituents showed smooth drug diffusion and protection against neurodegenerative disorders.

**Conclusion:**

This study indicates that *M. esculenta* extracts may hold significant therapeutic potential for neurodegenerative diseases, opening a path for potential therapeutic strategies.

## 1 INTRODUCTION

Alzheimer's disease (AD) is the most prevalent neurodegenerative disorder worldwide, affecting about 47 million people globally (Aarsland et al., 2021; Haque et al., 2018; Matthews et al., 2019; Stanciu et al., 2019). Clinically, it is characterized by memory and cognitive decline. Biochemically, it is characterized by extracellular plaques consisting of beta‐amyloid (Aβ) deposits and intracellular neurofibrillary tangles consisting of hyperphosphorylated tau protein aggregates. The AD pathogenesis is complex, and over the decades several hypotheses have been proposed. Among these, the cholinergic hypothesis is the earliest theory formulated. According to this, “when acetylcholinesterase (AChE), an enzyme that hydrolyzes acetylcholine, is overactive, it alters the structure of cholinergic synapses, depletes certain acetylcholine (ACh) receptors, kills ACh‐producing neurons, and reduces cholinergic neurotransmission” (Fu et al., 2023). ACh is a neurotransmitter that plays a critical role in memory and learning by transporting nerve impulses to muscle tissues. In AD, the activity of AChE increases, breaking down its substrate, Ach, and resulting in cognitive decline and memory loss (Wanleenuwat et al., 2019; Mathew et al., 2019). Compared to other Aβ‐associated proteins, AChE is a powerful amyloid‐promoting agent. Thus, modulating Ach levels or inhibiting AChE could represent a potential treatment for AD (Sinyor et al., 2020).

Chronic inflammation and oxidative stress synergistically contribute to neuronal damage and cognitive decline. In AD, oxidative stress is primarily instigated by mitochondrial dysfunction and the aberrant accumulation of redox‐active metals, leading to excessive reactive oxygen species (ROS) that damage lipids, proteins, and nucleic acids within neurons (Y. Zhao & Zhao, 2013; Huang et al., 2016). This oxidative imbalance not only facilitates amyloid beta (Aβ) plaque formation and tau hyperphosphorylation but also activates microglial cells, which sustain a pro‐inflammatory environment by releasing cytokines and further ROS, exacerbating neuronal damage (Kinney et al., 2018). Such a chronic neuroinflammatory state not only accelerates Aβ and tau pathologies but also establishes a vicious cycle where inflammation amplifies oxidative damage, ultimately leading to synaptic dysfunction and neuron loss (Chauhan & Chauhan, 2006; Liuzzi et al., 2023).

Currently, AD treatment is primarily symptomatic and has several limitations, including the side effects, which can significantly impact the quality of patients' lives and their caregivers (Jafari et al., 2018). Commercially approved acetylcholinesterase inhibitors, antioxidants, and anti‐inflammatory compounds, which are commonly used to treat AD, can cause nausea, diarrhea, vomiting, and muscle cramps (Chin et al., 2022). Given these limitations, there is a growing need for potent, natural, cost‐effective, and nontoxic treatment options. Natural sources of antioxidants and anti‐inflammatory compounds have been investigated as therapeutic strategies to disrupt this cycle (Kewlani et al., 2023). Mushrooms, particularly Morchella species, are emerging as promising candidates due to their rich secondary metabolites, which exhibit both antioxidant and anti‐inflammatory properties (Badshah et al., 2021).

Naturally available medicinal mushrooms have recently attracted much attention in several fields, including neurodegenerative disorders (Pohl et al., 2018). The presence of bioactive constituents in morel mushrooms may lead to the development of promising alternative therapeutic strategies (Loha et al., 2019; Wu et al., 2021). *Morchella esculenta*, verified name *Morchella esculenta* L. Pers (https://www.gbif.org/species/2594602), also known as the morel or true morel, is a fungus found in dense coniferous forests and mountainous regions worldwide, especially in Asia, the Himalayan Mountains, Europe, Mediterranean countries, and North America. In Pakistan's northern areas, *Morchella spp*. serves as a natural source of bioactive compounds. Powdered *Morchella* is advised to treat arthritis stomachache and as an emollient (Mahmood et al., 2011). Additionally, further studies on *M. esculenta* indicated that deproteinized polysaccharides (Sunil & Xu, 2022) from these mushrooms have moderate free radical scavenging activity and inhibitory function of AChE and butyrylcholinesterase (BChE), suggesting their potential role in treating neurodegenerative disorders (Badshah et al., 2021).

Despite the potential therapeutic benefits of morels, the pharmacological properties of *M. esculenta* are not fully understood. This study aims to fill this gap by conducting a comparative pharmacological analysis of the mycelia and fruiting body extracts of *M. esculenta*. Additionally, the study explored the potential of morels in AD treatment.

## 2 EXPERIMENTAL SECTION

### 2.1 Ethical statement

The institutional ethical review board of COMSATS University Islamabad (CUI), Abbottabad Campus, Pakistan, approved the study. The study participant's signed informed consent was obtained before the blood samples were collected.

### 2.2 Chemicals and reagents

Common chemicals, namely ethanol, methanol, sodium hydroxide, ferric chloride, lead acetate, HCl, chloroform, H_2_SO_4,_ PDA media, malt extract, bovine serum albumin, and DPPH (diphenyl‐1‐picrylhydrazyl), were taken from different commercially approved companies. While Mayer's and Wagner's reagents, Folin–Ciocalteu (FC) Reagent, AChE enzyme, and its substrate and reagents were obtained from Sigma Aldrich.

### 2.3 Sample collection

Northern regions of Pakistan, including Skardu, Malam Jabba, Krare Mansehra, and Thandiani, were visited to collect the ascocarps of morels. *Morchella esculenta* (Voucher CUHA‐351) fruiting bodies were collected. Microscopic examination was conducted using an Olympus X50 light microscope at 40X and 100X magnifications to observe diagnostic features, including ascospores, ascus, and paraphyses. Identification was carried out by systematically comparing the observed morphological traits with taxonomic descriptions available in the literature, particularly the works of S. Ali et al. (2021) and Kuo et al. (2012). The collected ascocarps underwent a distilled water wash and air drying. After drying, half of the samples were used for mycelia growth, and the remaining samples were ground to form powder with a mechanical blender and stored for further analysis (Su et al., 2020).

### 2.4 Mycelia's growth on solid media

#### 2.4.1 Media preparation and sterilization

Potato dextrose agar (PDA) was used to grow mycelia. In 1000 mL of distilled water, 39 g was dissolved to obtain PDA media (Gautam et al., 2019). Fruiting bodies of morels (1 g) were dissected by a sterilized surgical blade and placed on plated media. Plates were sealed with parafilm and placed in an incubator at 18°C for 2–3 days.

#### 2.4.2 Mycelia growth on liquid media

Light malt extract (LME) and PDA were used to prepare the suspension culture (Dang et al., 2018). A mycelial disc was cut using a sterile cork borer and placed in liquid media. Then, samples were placed on a magnetic stirrer for 2–3 weeks, and optical density was recorded and left till dried.

### 2.5 Methanolic extract preparation

Dried fruiting bodies and mycelia of *M. esculenta* samples were crushed to obtain fine powder. Both samples (1 g) were added into separate centrifuge tubes with 15 mL methanol and placed in an orbital shaker for 24 h. After 1 day, all samples underwent vortexing and centrifugation (10 min at 8000 rpm). Then, the supernatant was collected in a 50 mL centrifuge tube, and centrifugation was repeated three times. Samples were left under a fan for 2–3 days till extracts were completely dried. After drying, crude extracts were obtained, and their methanolic stock solution (100 µg/mL) was prepared for further analysis.

#### 2.5.1 Percentage yield of the extracts

The following formula was applied to estimate the percentage yields for all extracts :

$$\mathrm{Percentage}\;\mathrm{yield}\;=\left(\frac{\mathrm{Actual}\;\mathrm{yield}}{\mathrm{Theoretical}\;\mathrm{yield}}\right)\times \;100$$

### 2.6 Qualitative phytochemical analysis

The methanolic extracts of *M. esculenta* were tested for phytochemical screening using standard methods (Shaikh et al., 2020). To test phenolic compounds, alkaloids, flavonoids, glycosides, and saponins in *M. esculenta* methanolic extracts, ferric chloride test (Shaikh et al., 2020), Dragendroff's test (Tanzey et al., 2020), ammonia test (Greenlee et al., 2018), Salkowski's test, and sodium bicarbonate test (Delextrat et al., 2018) were performed, respectively.

### 2.7 Quantitative phytochemical analysis

#### 2.7.1 Total phenolic content

Folin–Ciocalteu reagent was used to detect the presence of polyphenols in *M. esculenta* extracts (Malek et al., 2019). FC reagent and Na₂CO₃ were added separately to the extracts. The mixture was incubated for 15–30 min at room temperature. The sample absorbance was measured at 650 nm by a spectrophotometer. Gallic acid was used as standard.

### 2.8 Antioxidant activities

#### 2.8.1 Diphenyl‐1‐picrylhydrazyl assay

Free radical scavenging activity was assessed by performing a DPPH assay (Yeo et al., 2019) Briefly, DPPH solution (3 mg/25 mL) was prepared. Multiple concentrations (1.26–50 µg/mL) of *M. esculenta* extracts and standard gallic acid were used for analysis. DPPH + ethanol were the negative controls. After adding 1 mL of DPPH to *M. esculenta* extracts and gallic acid dilutions, samples were incubated at room temperature in the dark for 1 h. At 517 nm, absorbance was detected, and by using the following formula, the free radical scavenging activity of all samples was determined:

$$\begin{array}{ccc} & & \mathrm{Percentage}\;\mathrm{radical}\;\mathrm{scavenging}\;\mathrm{activity}\;=\\ & & \frac{\mathrm{Absorbance}\;\mathrm{of}\;\mathrm{control}\;-\;\mathrm{Absorbance}\;\mathrm{of}\;\mathrm{sample}}{\;\mathrm{Absorbance}\;\mathrm{of}\;\mathrm{control}}\times 100 \end{array}$$

#### 2.8.2 2,2′‐casino‐bis(3‐ethylbenzothiazoline‐6‐sulfonic acid) assay

The 2,2′‐casino‐bis(3‐ethylbenzothiazoline‐6‐sulfonic acid) (ABTS) assay was performed to analyze the antioxidant activity of the methanolic extract of *M. esculenta*. ABTS assay was performed (Sridhar et al., 2019). A stock solution of 7 mM ABTS and potassium per‐sulfate was prepared in ultra‐pure water. Incubation for 14 h was done in the dark at room temperature. 1 mL of ABTS was diluted with 60 mL of methanol to dilute the solution. Serial dilutions were prepared (1.56–100 µg/mL). Gallic acid was used as a standard. The absorbance was detected at 734 nm.

### 2.9 Determination of anti‐inflammatory activity

#### 2.9.1 Inhibition of protein denaturation method with bovine serum albumin

The bovine serum albumin (BSA) denaturation assay was performed to assess anti‐inflammatory activity (Harrabi et al., 2018). Briefly, serial dilutions of *M. esculenta* extracts (25–400 µg/mL) were prepared along with the standard drug, aspirin. Note that 1% BSA solution was prepared and added to all serial dilutions. For 20 min, samples underwent incubation at 37°C in a water bath and then a second incubation at 57°C. At 660 nm, absorbance was measured after cooling samples at room temperature, with PBS + BSA serving as the negative control. The percentage of BSA denaturation inhibition was calculated using the following formula:

$$\begin{array}{ccc} & & \mathrm{Percentage}\;\mathrm{inhibition}=\\ & & \frac{{\mathrm{Control}}^{\prime }s\;\mathrm{absorbance}\;-\;\mathrm{Test}\;{\mathrm{sample}}^{\prime }s\;\mathrm{absorbance}}{{\mathrm{Control}}^{\prime }s\;\mathrm{absorbance}}\\ & & \times \;100 \end{array}$$

#### 2.9.2 Human red blood cell membrane stabilization test

The human red blood cell (HRBC) membrane stabilization assay was performed to assess the anti‐inflammatory activity. Erythrocyte suspension was prepared to perform the HRBC membrane stabilization test. Fresh human blood (3 mL) was drawn from a healthy individual who did not take any NSAIDs in the last 2 weeks. At a speed of 3000 rpm, the whole blood was centrifuged for 10 min, followed by a saline solution wash. This step was repeated three times. The supernatant obtained after the third centrifugation was clear and discarded. Blood volume was measured. Normal saline was added to obtain a 10% v/v solution. Multiple serial concentrations (6.25–400 µg/mL) of *M. esculenta* extracts and a standard drug, aspirin, were prepared. 1 mL of RBC suspension was added to each concentration of *M. esculenta* samples as well as to aspirin. The samples were incubated for 30 min at 56°C. Then, samples were centrifuged for 5 min at 2500 rpm. Saline + RBC suspension was used as a negative control. The percentage stabilization of the membrane was determined by the following formula after measuring absorbance at 560 nm.

$$\begin{array}{ccc} & & \mathrm{Percentage}\;\mathrm{protection}\;=\\ & & \left(100\;-\;\left[\left.\frac{\mathrm{Absorbance}\;\mathrm{of}\;\mathrm{the}\;\mathrm{test}\;\mathrm{sample}}{\mathrm{Absorbance}\;\mathrm{of}\;\mathrm{the}\;\mathrm{control}}\right]\right.\right)\times 100 \end{array}$$

### 2.10 Acetylcholinesterase inhibition assay

AChE inhibition assay was performed to analyze AChE inhibition activity of extracts (Jin et al., 2020). Briefly, 5 µL of enzyme solution (0.05 units/mL), 55 µL of PBS, and 20 µL of *M. esculenta* extracts were mixed and incubated at 37°C for 15 min in a microplate. DNTB (10 µL; 5 mM) and acetylthiocholine chloride (ATCCl; 10 µL) were added to each well. A colored product of 5‐thio‐2‐nitrobenzoate anion was formed, representing the hydrolysis of ATCCI. After 10 min of incubation, absorbance was recorded at 410 nm. Donepezil was used as a standard drug. The percentage of AChE inhibition was determined using the following formula:

$$\begin{array}{ccc} \mathrm{Percentage}\;\mathrm{inhibition} & = & \left(1\;-\;\frac{{\mathrm{Sample}}^{\prime }s\;\mathrm{absorbance}}{{\mathrm{Control}}^{\prime }s\;\mathrm{absorbance}\;}\right)\\ & & \times 100 \end{array}$$

### 2.11 Enzyme kinetics

For kinetic analysis of AChE activity and its suppression in the presence of the inhibitors KMFB (4.5, 5.5, and 7.5 µg/mL) and KMM (7.0, 8.2, and 9.5 µg/mL), different doses of the substrate (0.6, 1.2, and 1.8 mM) were used. Donepezil (17.4, 20.4, and 22.4 µg/mL) was the reference drug (Djeujo et al., 2022). Spectrophotometric measurements were made for a total of 10 min at a wavelength of 405 nm to investigate the kinetics of AChE hydrolysis with and without an inhibitor, and 1‐min intervals were used to capture the absorbance values. *K*i, *V*max, and *K*m values were the kinetic parameters that were calculated using Lineweaver–Burk and Dixon plots.

### 2.12 Molecular docking and ADMET analysis

The presence of ligands in the receptor's binding pocket was evaluated by molecular docking (Santos et al., 2019). After the docking has been performed, MOE stores the docking scores, Root mean square deviation (RMSD) values, and other interaction energy data in a database (Aziz et al., 2022). Chemical absorption, distribution, metabolism, excretion and toxicity (ADMET) analysis was also performed. The QikProp tool of Maestro was used to predict the physicochemical properties of the compounds of *M. esculenta* fruiting bodies and mycelia, including their molecular weight, ADMET qualities, and hydrogen bond donors and acceptors (Amengor et al., 2022).

### 2.13 Statistical analysis

The data were collected in triplicate readings for statistical analysis. Statistical analysis was conducted using GraphPad Prism 7.0. Nonlinear regression determined IC_50_ values for dose‐response studies, while enzyme kinetics were analyzed with Lineweaver–Burk and Dixon plots using linear regression to obtain kinetic parameters. A two‐way analysis of variance compared compound effects across concentrations, with Tukey's post hoc test identifying significant group differences. Graphical evaluations were conducted using Microsoft Excel 2013. A *p*‐value of <0.05 was considered statistically significant.

## 3 RESULTS

### 3.1 Percentage yield of extracts

All four samples yielded significantly elevated amounts of extracts (Table 1). KMFB (44.61%) showed the highest percentage yield along with its mycelia sample (KMM, 35.36%) (Table 1).

**TABLE 1:** Percentage yield of all *Morchella esculenta* extracts.

Fruiting body extract	Percentage yield (%)	Mycelia extract	Percentage yield (%)
SKFB	19.23	SKM	11.78
MJFB	17.68	MJM	18.45
KMFB	44.61	KMM	35.36
TFB	39.32	TM	26.13

*Note*: Data represent percentage yield of all samples of *M. esculenta* fruiting body and mycelia collected from northern regions of Pakistan.

Abbreviations: KMFB, Krair Mansehra fruiting body; KMM, Krair Mansehra mycelia; MJFB, Malam Jaba fruiting body; MJM, Malam Jaba mycelia; SKFB, Skardu sample fruiting body; SKM, Skardu sample mycelia; TFB, Thandiani fruiting body; TM, Thandiani mycelia.

### 3.2 Estimated phytoconstituents qualitative analysis

Phytochemical screening was performed to identify secondary metabolites in the fruiting body and mycelia of *M. esculenta*. Table 2 shows the changes in samples after performing phytochemical screening tests. The tests suggested that all eight extracts of the fruiting body and mycelia contain flavonoids, phenols, alkaloids, glycosides, and saponins (Table 2).

**TABLE 2:** Secondary metabolites in different extracts of *Morchella esculenta* fruiting body collected from northern regions of Pakistan.

			Results			
Phytochemicals	Characteristics		SKFB/SKM	MJFB/MJM	KMFB/KMM	TFB/TM
Phenol	Dark‐green coloration indicates the presence of phenol	Fruiting body	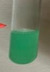 ++++	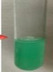 ++++	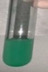 +++	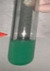 +++
		Mycelia	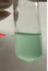 ++++	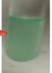 ++	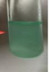 +++	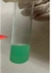 +++
Flavonoids	Intense yellow color which become colorless upon addition of few drops of diluted acid	Fruiting body	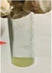 ++++	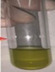 ++++	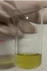 ++++	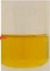 +++
		Mycelia	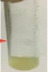 ++++	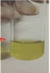 ++	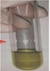 +++	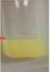
Alkaloids	Orange‐red precipitates indicate the presence of Alkaloids	Fruiting body	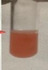 ++++	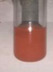 ++++	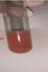 +++	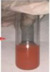 +++
		Mycelia	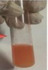 ++++	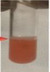 ++	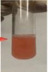 +++	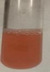 +++
Saponins	White precipitates indicate the presence of saponins	Fruiting body	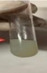 ++++	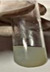 ++++	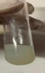 +++	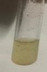 ++
		Mycelia	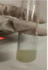 ++++	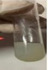 ++	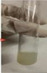 +++	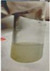 +++
Glycosides	Reddish brown color indicates the presence of glycosides	Fruiting body	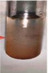 ++++	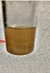 ++++	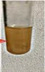 +++	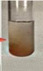 ++
		Mycelia	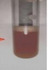 ++++	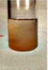 ++	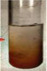 +++	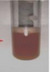 +++

*Note*: +: present, ++: moderately present, and +++: highly present.

Abbreviations: SKFB/SKM, fruiting body and mycelia of *M. esculenta* Skardu sample; MJFB/MJM, fruiting body and mycelia of *M.esculenta* Malam jaba sample; KMFB/KMM, fruiting body and mycelia of *M. esculenta* Krair Mansehra sample; TFB/TM, fruiting body and mycelia of *M. esculenta* Thandiani sample.

### 3.3 Phytochemicalsanalysis (quantitative)

#### 3.3.1 Estimation of total phenolic content

Total phenolic content (TPC) was measured by standard curve, and final values of TPC were presented as mg gallic acid equivalent/100g based on dry weight (mg GAE/100g, DW). The calculated dry weight of SKFB, MJFB, KMFB, and TFB was 386.6 ± 2.6, 220.8 ± 1.02, 284.5 ± 1.3, and 354.0 ± 1.7 mg, respectively, while that of SKM, MJM, KMM, and TM was 311.0 ± 1.8, 176.3 ± 3.9, 245.8 ± 5.01, and 292.8 ± 3.6 mg, respectively (Table 3). *Morchella esculenta* showed the trend from highest to lowest TPC as follows: SKFB/SKM > TFB/TM > KMFB/KMM > MJFB/MJM.

**TABLE 3:** Total phenolic content of *Morchella esculenta* fruiting bodies and mycelia.

Extract	Fruiting body (mg)	Mycelia (mg)
SKFB/SKM	386.6 ± 2.6	311.0 ± 1.8
MJFB/MJM	220.8 ± 1.02	176.3 ± 3.9
KMFB/KMM	284.5 ± 1.3	245.8 ± 5.01
TFB/TM	354.0 ± 1.7	292.8 ± 3.6

*Note*: Data represent mean values ± standard error of three replicates. Each mean value followed by a specific letter represents a significant difference at *p* < 0.05.

Abbreviations: SKFB/SKM, fruiting body and mycelia of *M. esculenta* Skardu sample; MJFB/MJM, fruiting body and mycelia of *M.esculenta* Malam jaba sample; KMFB/KMM, fruiting body and mycelia of *M. esculenta* Krair Mansehra sample; TFB/TM, fruiting body and mycelia of *M. esculenta* Thandiani sample.

### 3.4 Oxidative stress relieving characteristics of *M. esculenta* extracts

#### 3.4.1 Antioxidant effects of *M. esculenta* extracts via DPPH assay

**FIGURE 1:**
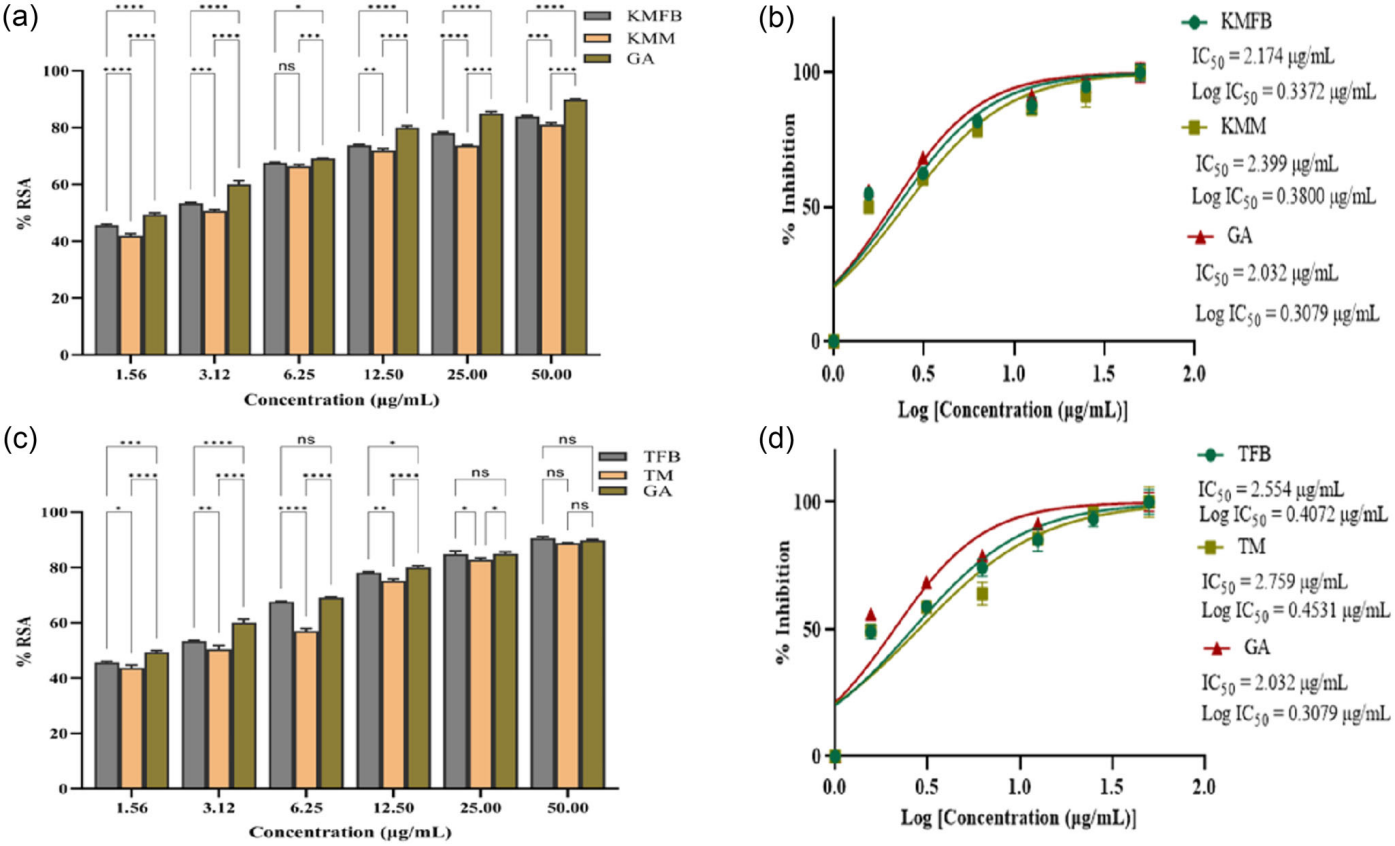
(a and c) The diphenyl‐1‐picrylhydrazyl (DPPH) radical scavenging activity of methanolic extracts of *M. esculenta* fruiting body and mycelia (Krair Mansehra fruiting body [KMFB]/Krair Mansehra mycelia [KMM] and Thandiani fruiting body [TFB]/Thandiani mycelia [TM]) samples at different concentrations compared to Gallic acid as standard, along with their corresponding error amount as a percentage. (b and d) IC_50_ graph shows the DPPH radical scavenging activity of varying concentrations of methanolic extracts of selected samples along with gallic acid. Statistical significance is represented as follows: ^*****^ *p* < 0.0001, ^***^ *p* < 0.001, ^**^ *p* < 0.01, ^*^ *p* < 0.05, and ^ns^ *p* ≥ 0.05. GA, gallic acid; ns, nonsignificant.

**FIGURE 2:**
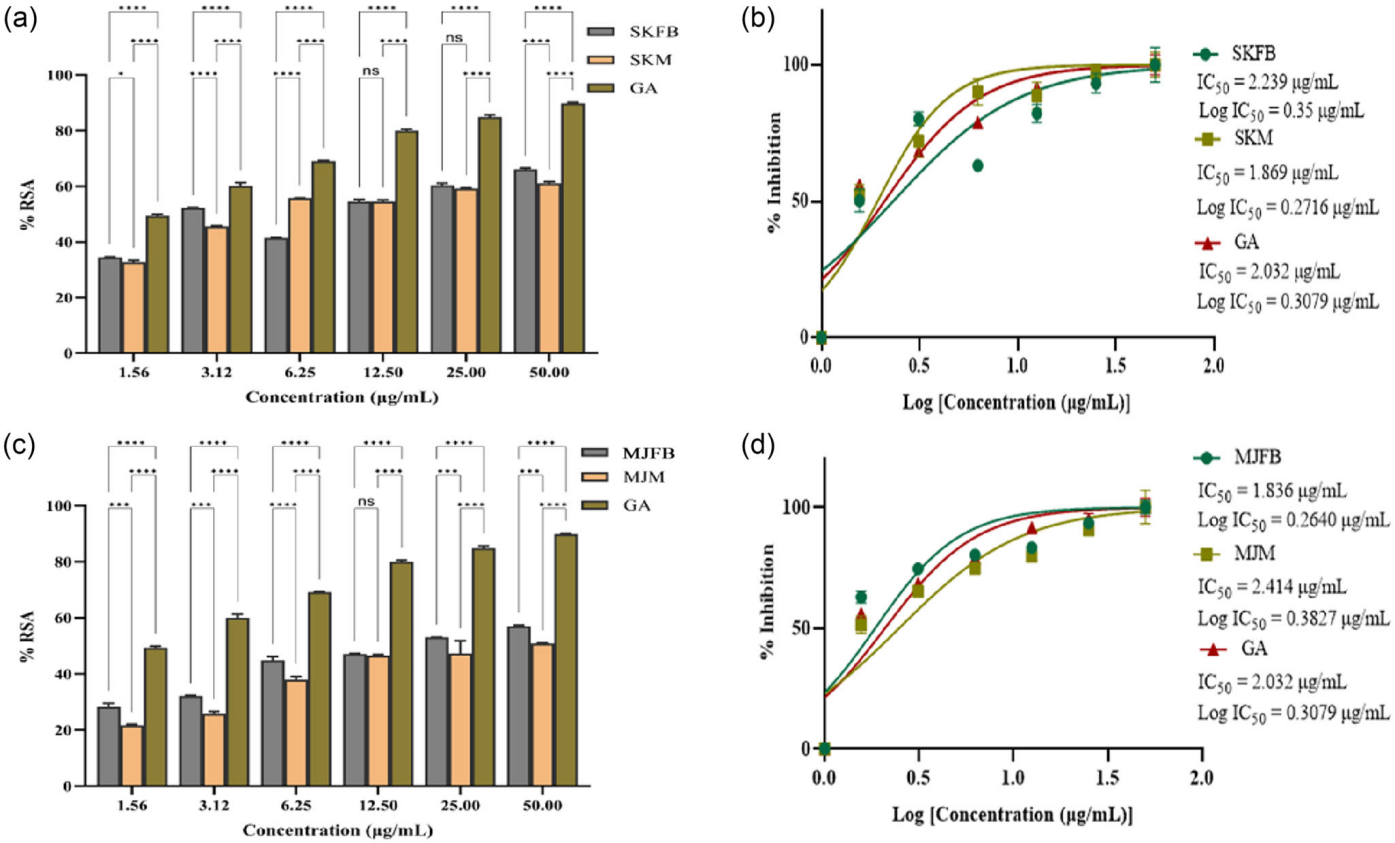
(a and c) The diphenyl‐1‐picrylhydrazyl (DPPH) radical scavenging activity of methanolic extracts of both fruiting body and mycelia of *Morchella* (Skardu sample fruiting body [SKFB]/Skardu sample mycelia [SKM] and Malam Jaba fruiting body [MJFB]/Malam Jaba mycelia [MJM]) sample at different concentrations in relation to Gallic acid as standard along with their respective error amount as a percentage. (b and d) IC_50_ graph shows the DPPH radical scavenging activity of different concentrations of methanolic extracts along with the standard gallic acid. Statistical significance is represented as follows: ^*****^ *p* < 0.0001, ^***^ *p* < 0.001, ^**^ *p* < 0.01, ^*^ *p* < 0.05, and ^ns^ *p* ≥ 0.05. GA, gallic acid; ns, nonsignificant.

Distinct serial dilutions of *M. esculenta* extracts (SKFB/SKM, MJFB/MJM, KMFB/KMM, and TFB/TM) treated with free radical molecules (DPPH) depicted their free radical scavenging activity. Figures 1 and 2 show the free radical scavenging activities of methanolic extracts of *M. esculenta*. Among the extractives, TFB/TM exhibited significant antioxidant activity (Figure 2c). The radical scavenging activity of fruiting body extracts, SKFB, MJFB, KMFB, and TFB, at 50 µg/mL, showed 65 ± 1.06%, 56 ± 2.08%, 80 ± 0.43%, and 85 ± 1.58% inhibition, respectively, along with gallic acid exhibiting 81 ± 0.89% antioxidant activity (Figures 1a,c and 2a,c). Alternatively, SKM, MJM, KMM, and TM samples at 50 µg/mL concentration showed 59%, 50%, 78%, and 82% scavenging activity, respectively. The free radical scavenging activity of different extracts and gallic acid was observed in the following order: TFB/TM > GA > KMFB/KMM > SKFB/SKM > MJFB/MJM. The IC_50_ values indicated that the methanolic extract of MJFB and SKM had the lowest IC_50_ of 1.836 ± 0.08 and 1.869 ± 0.60 µg/mL, respectively (Figure 1b,d).

#### 3.4.2 Antioxidant effects of *M. esculenta* extracts via ABTS assay

**FIGURE 3:**
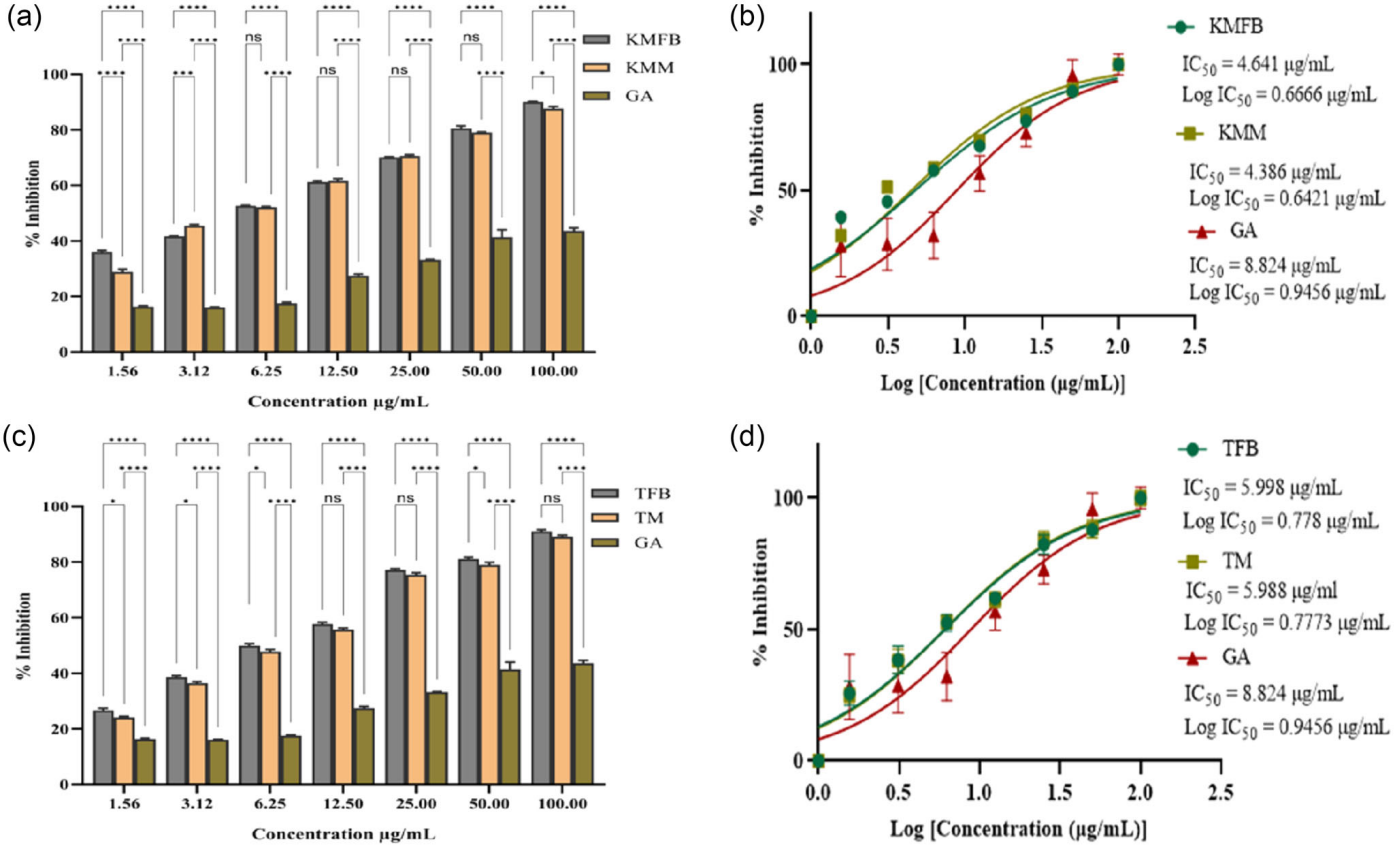
(a and c) Bar plots showing 2,2′‐casino‐bis(3‐ethylbenzothiazoline‐6‐sulfonic acid) (ABTS) radical scavenging activity of methanolic extracts of *M. esculenta*, fruiting bodies and mycelia (Krair Mansehra fruiting body [KMFB]/Krair Mansehra mycelia [KMM] and Thandiani fruiting body [TFB]/Thandiani mycelia [TM]), at different concentrations in comparison to gallic acid. (b and d) IC_50_ values graph showing ABTS radical scavenging activity of *M. esculenta* along with gallic acid, used as standard. Statistical significance is represented as follows: ^*****^ *p* < 0.0001, ^***^ *p* < 0.001, ^**^ *p* < 0.01, ^*^ *p* < 0.05, and ^ns^ *p* ≥ 0.05. GA, gallic acid; ns, nonsignificant.

**FIGURE 4:**
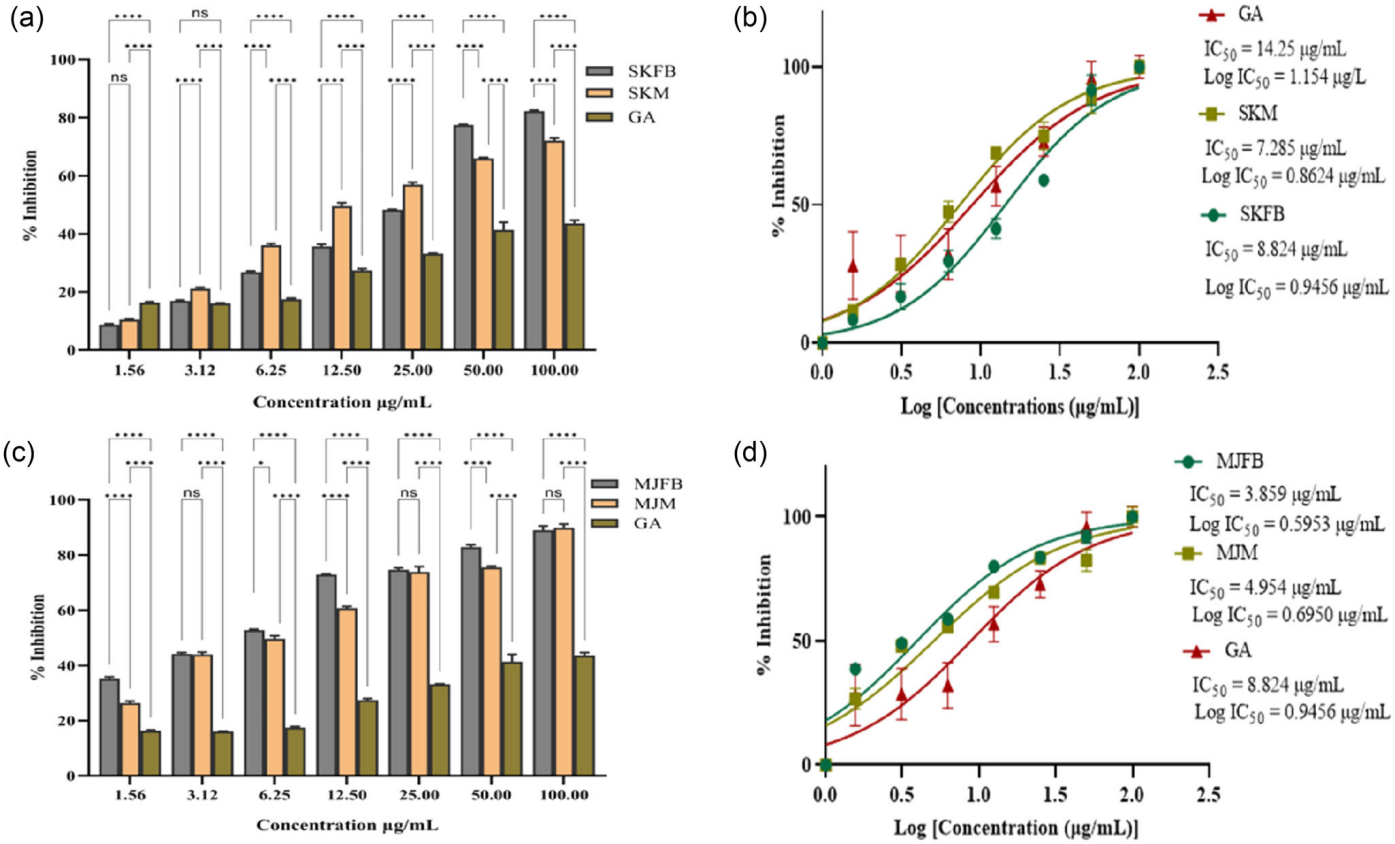
(a and c) Bar plots showing 2,2′‐casino‐bis(3‐ethylbenzothiazoline‐6‐sulfonic acid) (ABTS) radical scavenging activity of *M. esculenta* extracts (Skardu sample fruiting body [SKFB]/Skardu sample mycelia [SKM] and Malam Jaba fruiting body [MJFB]/Malam Jaba mycelia [MJM]) at different concentrations in comparison to gallic acid. (b and d) IC_50_ graph depicts the ABTS radical scavenging activity of varied concentrations of *M. esculenta* extracts in addition to the standard gallic acid. Statistical significance is represented as follows: ^*****^ *p* < 0.0001, ^***^ *p* < 0.001, ^**^ *p* < 0.01, ^*^ *p* < 0.05, and ^ns^ *p* ≥ 0.05. GA, gallic acid; ns, nonsignificant.

In the ABTS radical scavenging experiment, a dose‐response relationship was observed. All the extracts of *M. esculenta* showed maximum scavenging activity at 100 µg/mL concentration; on the other hand, gallic acid (standard) showed 44% scavenging potential (Figures 3 and 4). The ABTS assay showed antioxidant activity in the following order: MJFB/MJM > KMFB/KMM > SKFB/SKM ≥ TFB/TM. Figures 3 and 4 show the IC_50_ values of all *M. esculenta* extracts against ABTS radicals. The extracts of *M. esculenta* had remarkably more substantial potential for ABTS radical scavenging. Methanolic extracts of MJFB and KMM showed IC_50_ values of 3.859 ± 0.006 and 4.386 ± 0.00034 µg/mL, respectively, observed lesser than their standard gallic acid (8.824 µg/mL) (Figures 3d and 4b). It was found that these samples are potent ABTS radical scavengers, with a significant difference from all the samples of fruiting bodies of *M. esculenta* and gallic acid, as evidenced in nonlinear regression analysis, highlighting the antioxidant therapeutic potency of *M. esculenta* extracts. KMFB and TFB samples exhibited strong antioxidant potential with nonsignificant differences. Similarly, MJM, KMM, and TM showed the highest scavenging activity with negligible difference except for the gallic acid, which contained many phytochemicals as compared to the standard (Figures 3 and 4).

### 3.5 Unlocking therapeutic property of *M. esculenta* extracts as inflammatory inhibitors

#### 3.5.1 Inhibition of BSA protein denaturation

**FIGURE 5:**
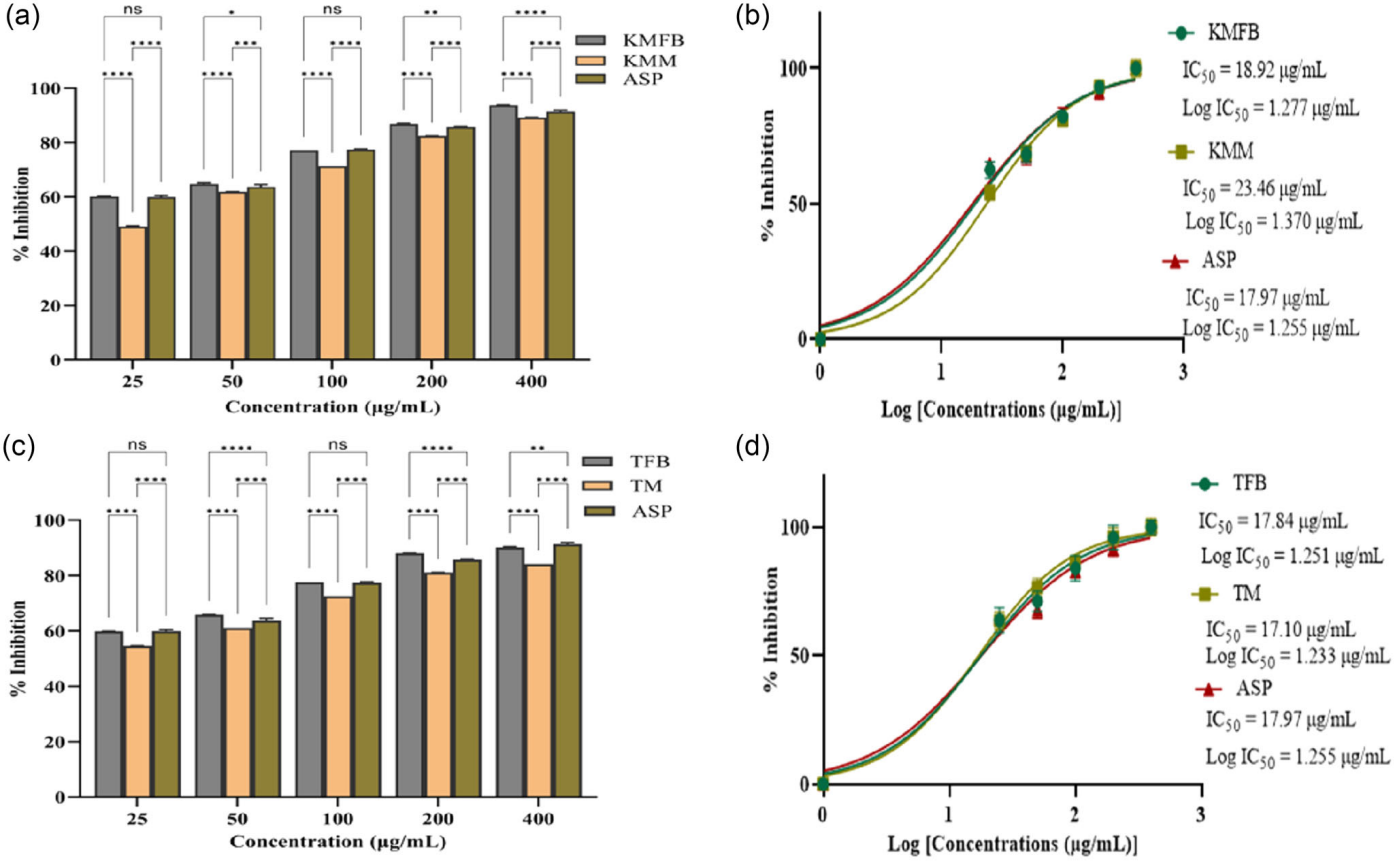
(a and c) Protein denaturation inhibition by different concentrations of *M. esculenta* (Krair Mansehra fruiting body [KMFB]/Krair Mansehra mycelia [KMM] and Thandiani fruiting body [TFB]/Thandiani mycelia [TM]) sample in comparison to aspirin (ASP) as standard, with respective error amount (%) (b and d) *M. esculenta* (Krair Mansehra fruiting body [KMFB]/KMM and TFB/TM) IC_50_ values for log concentrations. Statistical significance is represented as follows: ^*****^ *p* < 0.0001, ^***^ *p* < 0.001, ^**^ *p* < 0.01, ^*^ *p* < 0.05, and ^ns^ *p* ≥ 0.05. ns, nonsignificant.

**FIGURE 6:**
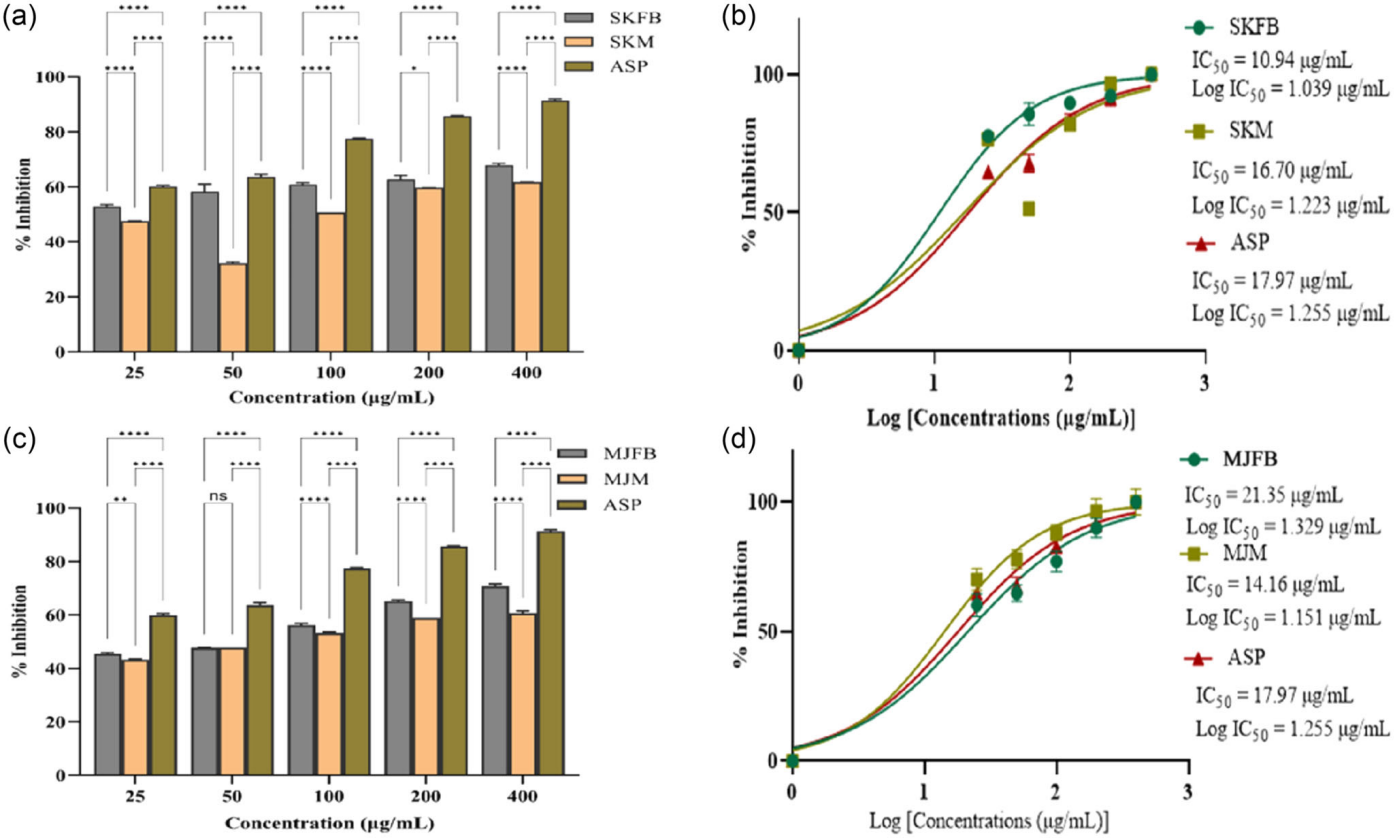
(a and c) Inhibition of protein denaturation by different concentrations of *Morchella esculenta* (Skardu sample fruiting body [SKFB]/Skardu sample mycelia [SKM] and Malam Jaba fruiting body [MJFB]/Malam Jaba mycelia [MJM]) sample in comparison to aspirin (ASP) as a standard drug along with respective error amount (%). (b and d) IC_50_ values for log of concentrations of *Morchella esculenta* (SKFB/SKM and MJFB/MJM). Statistical significance is represented as follows: ^*****^ *p* < 0.0001, ^***^ *p* < 0.001, ^**^ *p* < 0.01, ^*^ *p* < 0.05, and ^ns^ *p* ≥ 0.05. ns, nonsignificant.

The fruiting body of *M. esculenta* showed significantly higher BSA denaturation inhibition at 400 µg/mL. The extracts, KMFB/KMM and TFB/TM displayed significant inhibitory activity in comparison to the standard anti‐inflammatory drug, aspirin (Figures 5 and 6), proving that *M. esculenta* is more therapeutically beneficial in controlling inflammation as compared to ASP. The percentage inhibition of SKFB, MJFB, KMFB, and TFB at 400 µg/mL concentration was found as 63 ± 2.47%, 66 ± 0.14%, 95 ± 2.22%, and 86 ± 0.67%, respectively (Figures 5a,c and 6a,c). In contrast, SKM, MJM, KMM, and TM samples showed 58 ± 1.16%, 56 ± 1.43%, 85 ± 0.98%, and 78 ± 0.32% inhibition, respectively, at the same concentration (Figures 5a,c and 6a,c). The overall pattern of inhibition of BSA via *M. esculenta* samples was the following: KMFB/KMM > TFB/TM > MJFB > SKFB > SKM > MJM. The depicted IC_50_ values indicated that SKFB (10.94 ± 0.64 µg/mL) and MJM (14.16 ± 0.37 µg/mL) are potent inhibitors as compared to ASP (17.97 ± 0.44 μg/mL) (Figure 5b,d). Mycelia extract was found to have more significant scavenging potential than conventional medication. There is no significant difference between KMFB/KMM and TFB/TM samples of *M. esculenta* and aspirin in nonlinear regression analysis depicting nonsignificant antioxidant properties compared to the standard (Figure 6).

#### 3.5.2 Membrane stabilization of human RBCs

**FIGURE 7:**
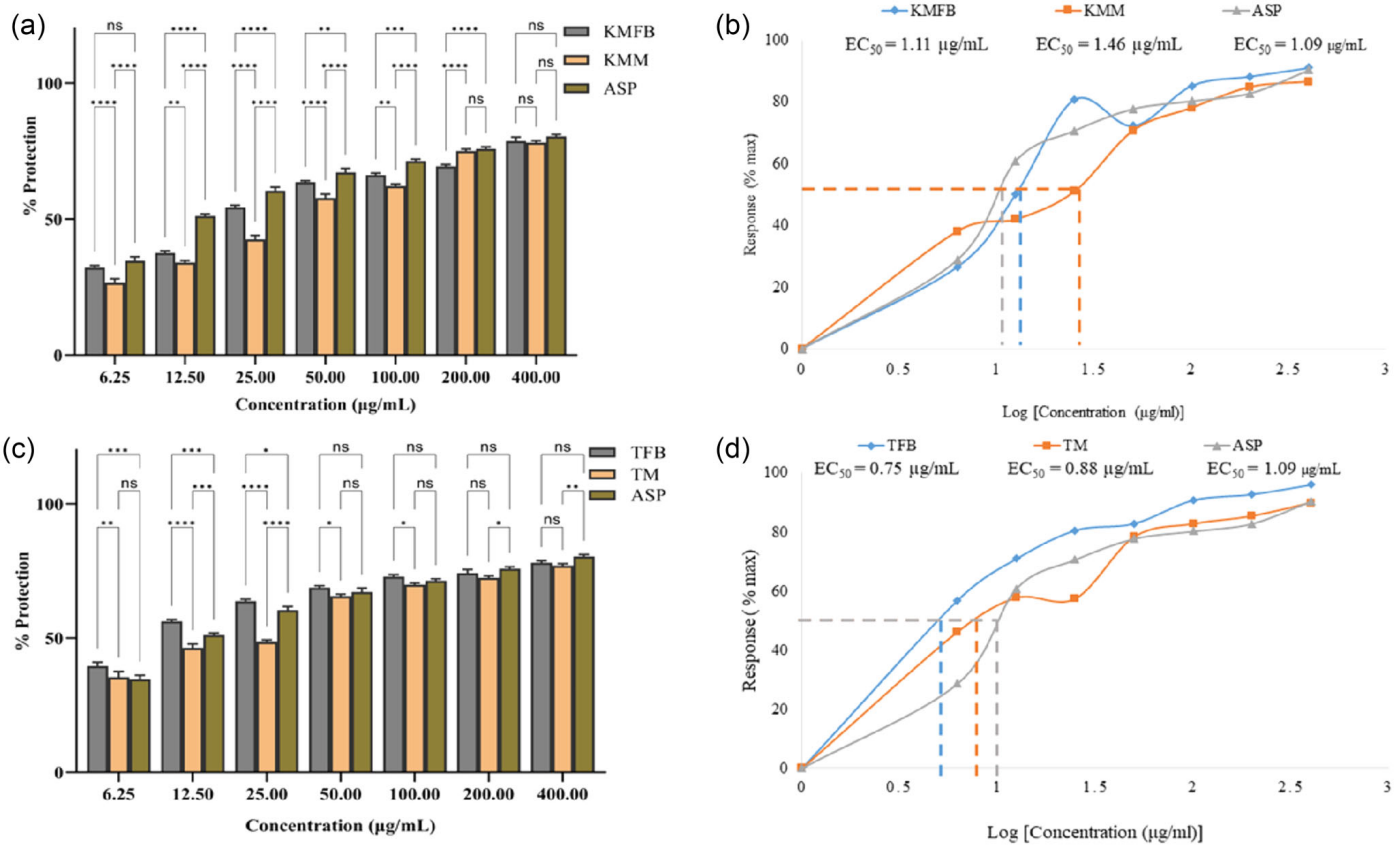
(a and c) Percentage protection of human red blood cells (HRBCs) by selected concentrations of methanolic extract of *Morchella esculenta* (Krair Mansehra fruiting body [KMFB]/Krair Mansehra mycelia [KMM] and Thandiani fruiting body [TFB]/Thandiani mycelia [TM]) sample in comparison with aspirin (ASP) along with respective error amount and (b and d). IC_50_ curve graph of *M. esculenta* (Krair Mansehra fruiting body [KMFB]/KMM and TFB/TM) sample. Statistical significance is represented as follows: ^*****^ *p* < 0.0001, ^***^ *p* < 0.001, ^**^ *p* < 0.01, ^*^ *p* < 0.05, and ^ns^ *p* ≥ 0.05. ns, nonsignificant.

**FIGURE 8:**
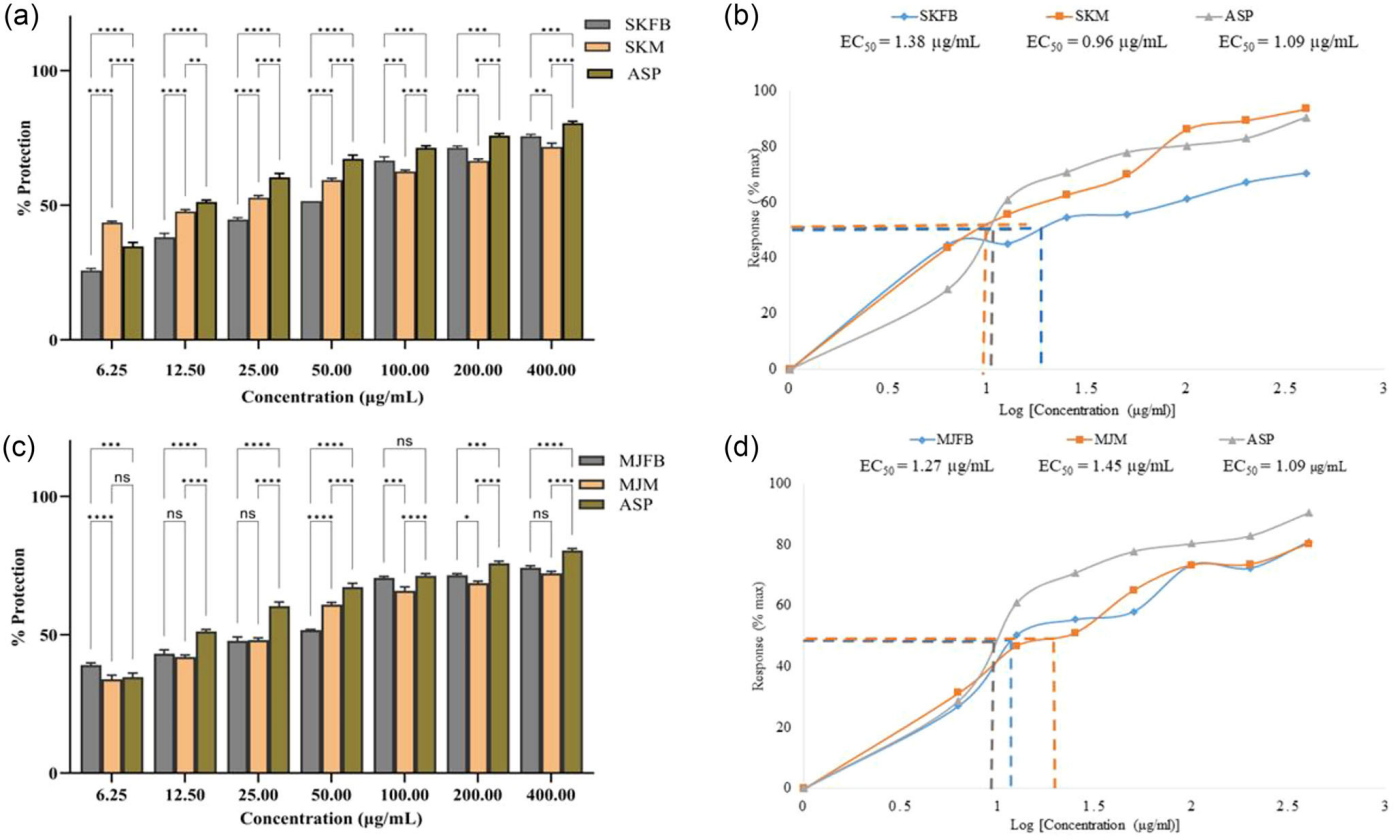
(a and c) Percentage protection of human red blood cells (HRBCs) by selected concentrations of methanolic extract of *Morchella esculenta* (Skardu sample fruiting body [SKFB]/Skardu sample mycelia [SKM] and Malam Jaba fruiting body [MJFB]/Malam Jaba mycelia [MJM]) sample in comparison with aspirin (ASP) along with respective error amount and (b and d). IC_50_ curve graph of *M. esculenta* (KMFB/KMM and TFB/TM) sample. Statistical significance is represented as follows: ^*****^ *p* < 0.0001, ^***^ *p* < 0.001, ^**^ *p* < 0.01, ^*^ *p* < 0.05, and ^ns^ *p* ≥ 0.05. ns, nonsignificant.

The potential of *M. esculenta* extracts to protect RBCs from hemolysis was analyzed to assess their effects on membrane stabilization. All the extracts ranging from 6.25 to 400 µg/mL concentrations significantly inhibited hemolysis of RBCs when compared with the aspirin. The fruiting body samples at higher concentrations showed the highest percentage of protection from hemolysis. SKFB, MJFB, KMFB, and TFB extracts at 6.25 µg/mL concentration showed 95 ± 0.04%, 83 ± 0.77%, 98 ± 0.06%, and 98 ± 0.03% membrane protection, respectively, while SKM, MJM, KMM, and TM samples showed 75 ± 0.11%, 84 ± 1.81%, 70 ± 1.43%, and 90 ± 0.93% scavenging activity, respectively (Figures 7 and 8). The percentage protection of all *M. esculenta* samples against hemolysis showed the following trend: KMFB ≥ TFB > SKFB > TM > MJM > MJFB > SKM > KMM. Extracts of *M. esculenta* showed significant differences with the same trend at a concentration of 400 µg/mL. For protection against heat‐induced hemolysis, extracts showed EC_50_ values of 1.38 ± 0.30, 1.27 ± 0.01, 1.11 ± 0.05, and 0.75 ± 0.03 µg/mL, respectively (Figures 7b,d and 8b,d). Aspirin showed an EC_50_ value of 1.09 ± 0.008 µg/mL. As compared to mycelia, fruiting body extracts significantly reduced the amount of heat‐induced hemolysis of HRBCs (Figures 7a,c and 8a,c). There is no significant difference in percentage hemolysis among all samples of the fruiting body of *M. esculenta* with aspirin in the nonlinear regression graph. In contrast, in percentage protection assay, KMFB/KMM and TFB/TM showed significant differences with aspirin, which indicates these have more scavenging activity than the standard at 400 µg/mL (Figure 8).

### 3.6 *M. esculenta* extracts as potential neuroprotective agents

#### 3.6.1 Estimation of acetylcholinesterase inhibition activity

**FIGURE 9:**
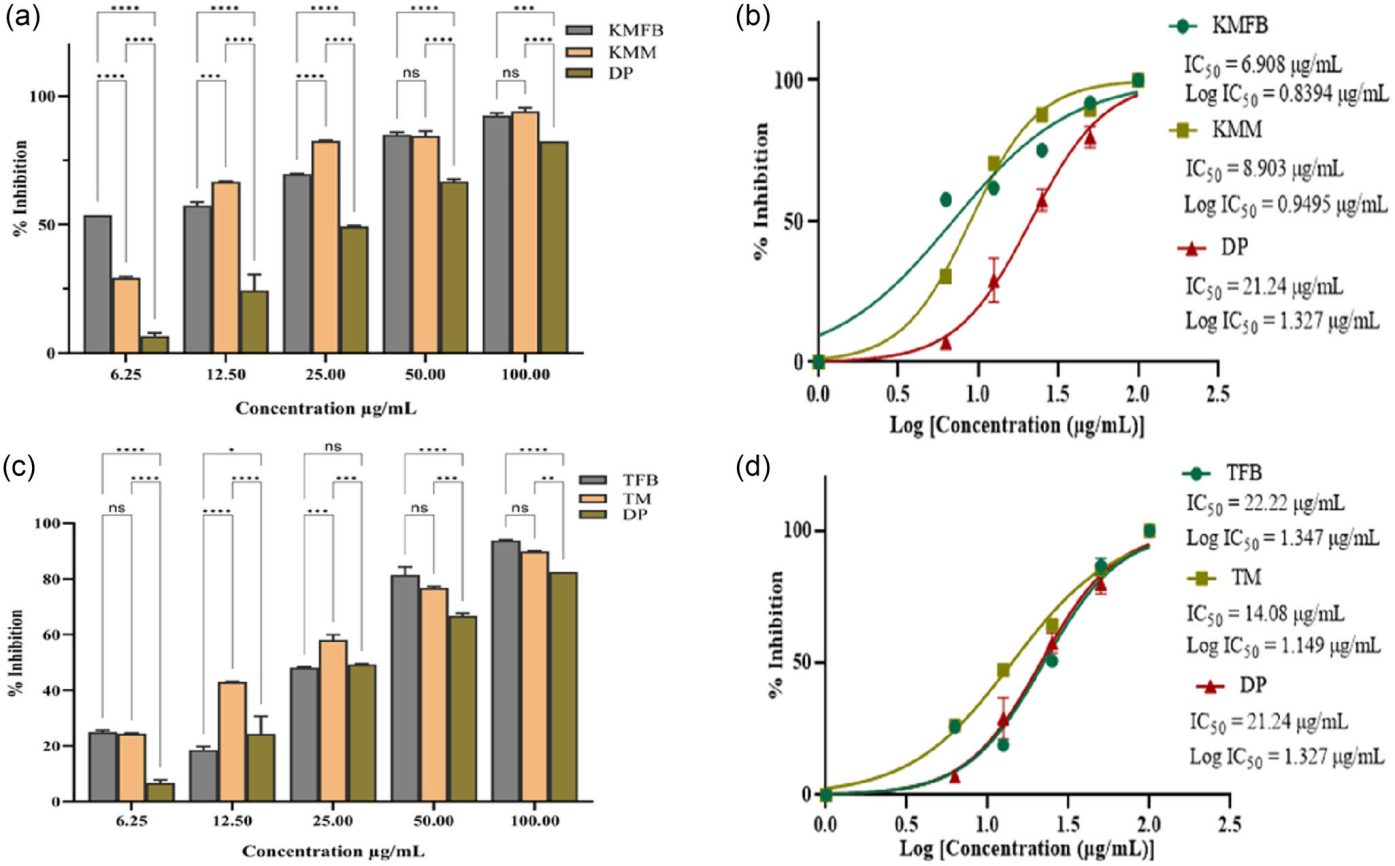
(a and c) Acetylcholinesterase (AChE) percentage inhibition by methanolic extract of *M. esculenta* (Krair Mansehra fruiting body [KMFB]/Krair Mansehra mycelia [KMM] and Thandiani fruiting body [TFB]/Thandiani mycelia [TM]) fruiting body and mycelia in comparison with Donepezil as standard drug. (b and d) IC_50_ values for log concentrations of *M. esculenta* extracts for estimating acetylcholinesterase inhibitory activity in comparison to aspirin. Statistical significance is represented as follows: ^*****^ *p* < 0.0001, ^***^ *p* < 0.001, ^**^ *p* < 0.01, ^*^ *p* < 0.05, and ^ns^ *p* ≥ 0.05. DP, Dixon plot; ns, nonsignificant.

**FIGURE 10:**
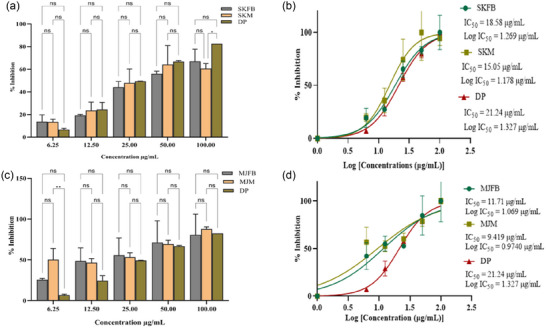
(a and c) Acetylcholinesterase (AchE) percentage inhibition by methanolic extract of *M. esculenta* (Skardu sample fruiting body [SKFB]/Skardu sample mycelia [SKM] and Malam Jaba fruiting body [MJFB]/Malam Jaba mycelia [MJM]) fruiting body and mycelia in comparison with Donepezil as standard drug. (b and d) Acetylcholinesterase inhibitory activity was estimated using IC_50_ values for log of concentrations of *M. esculenta* extracts and aspirin. Statistical significance is represented as follows: ^*****^ *p* < 0.0001, ^***^ *p* < 0.001, ^**^ *p* < 0.01, ^*^ *p* < 0.05, and ^ns^ *p* ≥ 0.05. DP, Dixon plot; ns, nonsignificant.

The AChE inhibition activity of all extracts was examined. The extracts showed a considerable inhibitory action. Compared to a typical anti‐inflammatory drug, both extracts showed potent anti‐inflammatory efficacy. Among the extracts, the fruiting body showed a more significant inhibition activity than donepezil. At 100 µg/mL concentration, SKFB, MJFB, KMFB, and TFB samples showed 74 ± 1.05%, 78 ± 0.17%, 92 ± 0.04%, and 93 ± 0.08% enzyme inhibition, respectively (Figures 9a,c and 10a,c). Mycelium extract of SKM, MJM, KMM, and TM samples at 100 µg/mL concentration showed 61 ± 1.27%, 86 ± 1.06%, 94 ± 0.03%, and 89 ± 1.15% enzyme inhibition activity, respectively (Figures 9a,c and 10a,c). The depicted IC_50_ values indicated that methanolic extracts of SKFB, MJFB, KMFB, and TFB samples were 18.58 ± 0.49, 11.71 ± 0.33, 6.908 ± 0.17, and 22.22 ± 0.39 µg/mL. At the same time, SKM, MJM, KMM, and TMM showed 15.05 ± 0.43, 9.419 ± 0.115, 8.903 ± 0.004, and 14.08 ± 0.002 µg/mL, respectively, which suggests a lower enzyme inhibition potential of fruiting body samples than mycelia and standard drugs (Figures 9b,d and 10b,d). There is a significant difference among all samples of the fruiting body of *M. esculenta* as compared to donepezil. At 100 µg/mL concentration, KMFB, TFB, KMM, and TM samples show higher anti‐enzymatic activity than other samples and donepezil, suggesting a high efficacy of KMFB and TFB samples. The same trend is observed for KMM and TM samples at the same concentration (Figures 9 and 10).

### 3.7 Enzyme kinetics analysis reveals distinct inhibitory mechanisms of *M. esculenta* methanolic extract and DP on AChE

**FIGURE 11:**
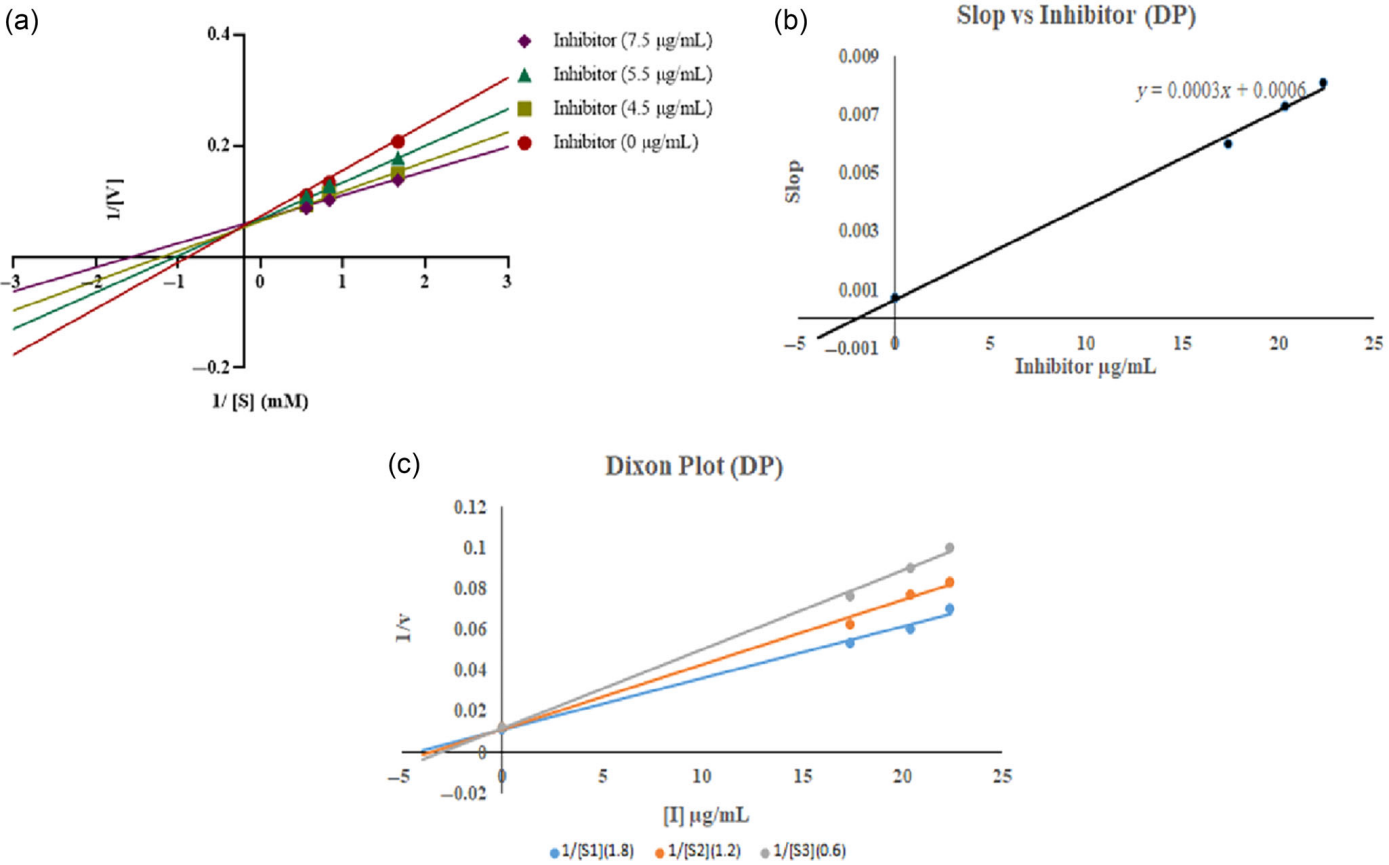
*M. esculenta* (Krair Mansehra fruiting body [KMFB]) kinetic analysis results: (a) Lineweaver‐Burk plot indicates inhibition of acetylcholinesterase (AChE) at varying concentrations of inhibitor (7.5, 5.5, and 4.5 µg/mL) and substrate at different concentrations (0.6, 1.2, and 1.8 mM, respectively); (b) inhibitor versus slope graph; and (c) Dixon plot.

**FIGURE 12:**
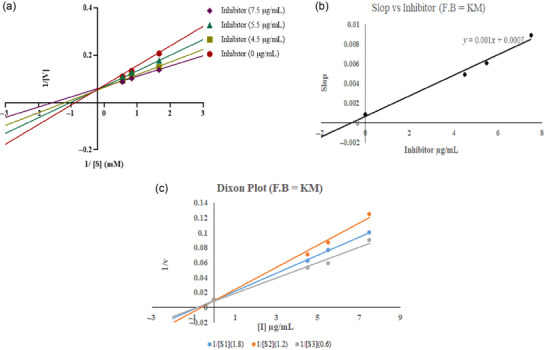
*M. esculenta* (Krair Mansehra mycelia [KMM]) kinetic analysis findings: (a) Lineweaver‐Burk plots showing acetylcholinesterase (AChE) inhibition at varying concentrations of inhibitor (9.5, 8.2, and 7.0 µg/mL) and substrate at different concentrations (0.6, 1.2, and 1.8 mM, respectively); (b) inhibitor versus slope graph; and (c) Dixon plot.

**FIGURE 13:**
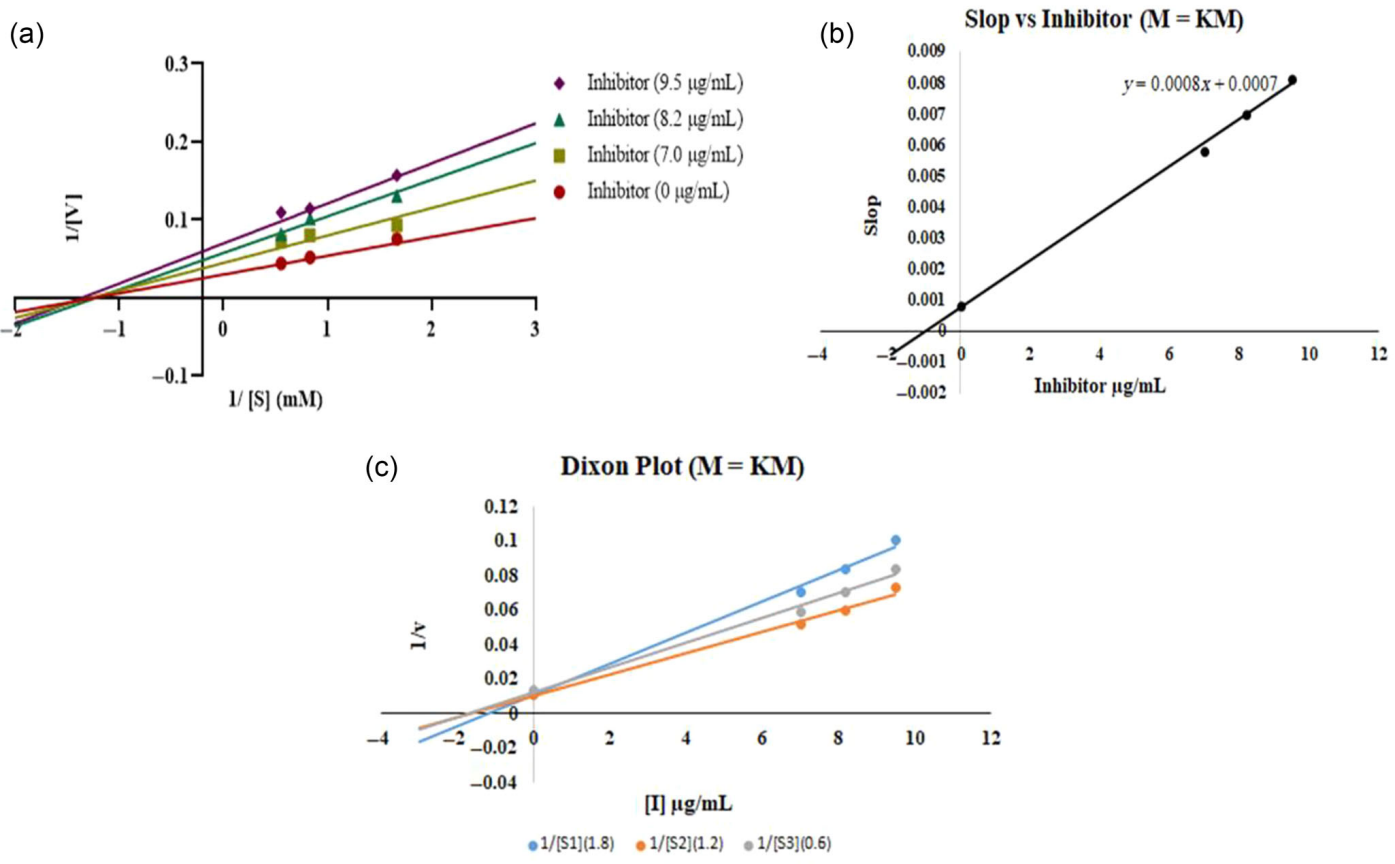
Kinetic analysis of reference (Donepezil) (a) Lineweaver–Burk plots for acetylcholinesterase (AChE) inhibition at various inhibitor (22.4, 20.4, and 17.4 µg/mL) and substrate (0.8, 1.2, and 1.5 mM, respectively) at varying concentrations.

The results of enzyme kinetics were analyzed by a Lineweaver–Burk double reciprocal plot of 1/*V* versus 1/[*S*]; “*V*” refers to velocity and “[*S*]” denotes substrate concentration (Figure 12). Figure 12 illustrates how *K*m, the Michaelis–Menten constant, and *V*max, the maximal enzyme activity, vary with time. In the *Y*‐axis (*Y*‐intercept = 1/*V*max), all trend lines for all concentrations of *M. esculenta* methanolic extract of KMFB at different concentrations where substrate intersected exhibits the same *V*max and intersect at a different point on the plot's *X*‐axis (*X*‐intercept = −1/K*m*), with different *K*m values. *K*i (inhibition constant) of the extract was calculated via Dixon plot and was *K*i = 0.7 µM (Figure 12b). *K*m and *V*max calculated values are displayed in Figure 12a,c, respectively. *K*m considerably decreased with increasing extract concentrations when varied extract concentrations were present, while *V*max was unchanged. This explains how KMFB binds to the active site of the enzyme and acts as a competitive inhibitor. While trend lines of KMM intersect at different points on the *Y*‐axis (*Y*‐intercept = 1/*V*max), having different *V*max and intersecting at different points on the *X*‐axis (*X*‐intercept = −1/*K*m) of the plot, revealing different values of *K*m. *K*i of the extract is 0.875 µM (Figure 13b), indicating that *K*m values are unaffected, and *K*m is also reduced, depicting mixed inhibition. On the other hand, all trend lines of different concentrations of DP (Figure 11) at the substrate's different concentrations showed similar behavior on both axes as KMM does with *K*i = 2 µM. This is because KMM and DP are noncompetitive AChE inhibitors that bind to the allosteric region of the substrate and to the enzyme's targeted site, regardless of any bound substrate.

### 3.8 Molecular docking, Physicochemical and ADMET analysis

**FIGURE 14:**
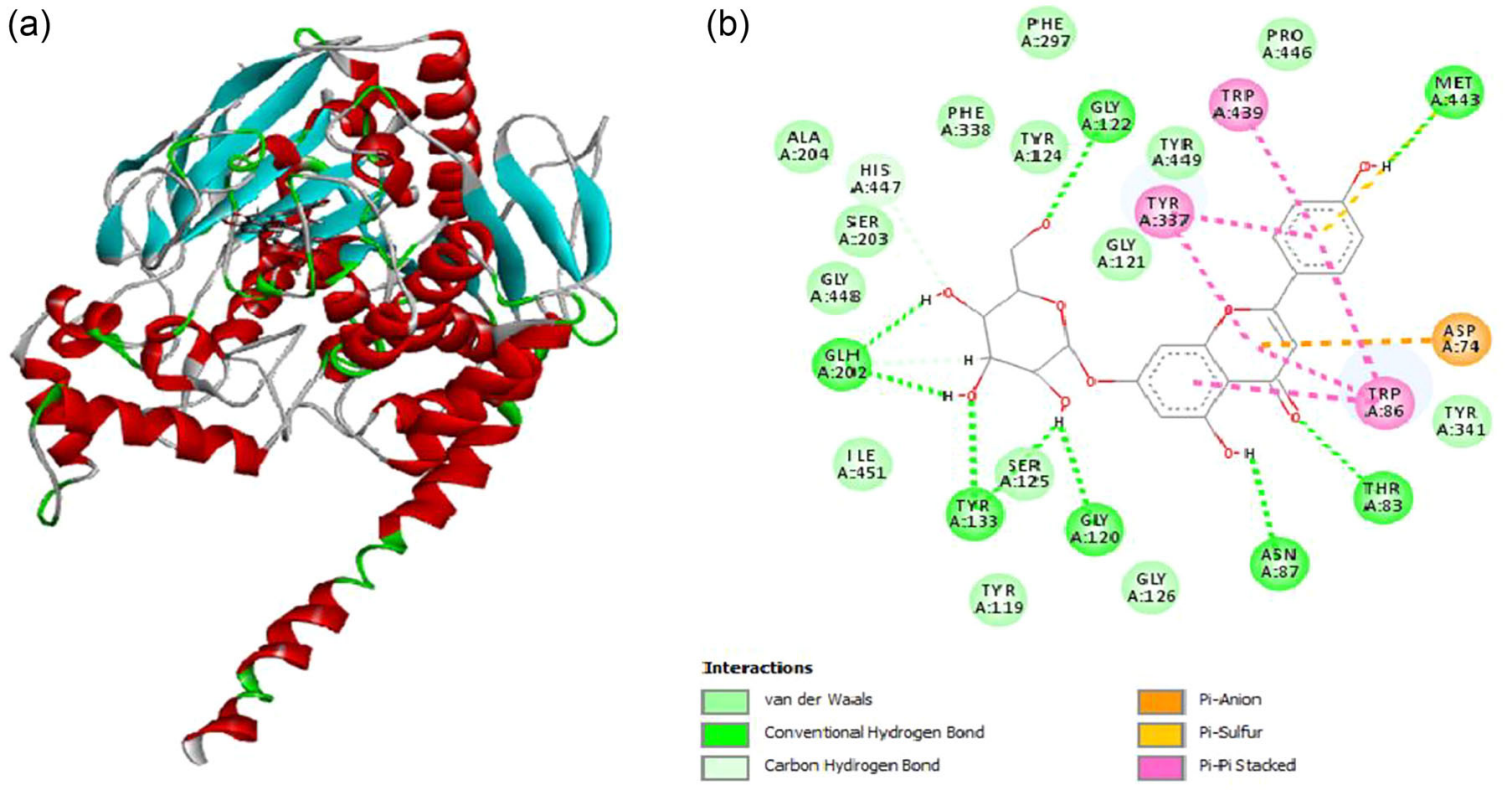
(a) Three‐dimensional (3D) structure of AChE (PDB entry: 4BDT). (b) Binding interactions of *M. esculenta* compound (Apigenin‐7‐O‐glucoside with amino acid residues of AChE depicting hydrogen bonding, pi‐pi interactions, vander waals interactions.

The interactions of M. esculenta bioactive constituents and donepezil with AChE (PDB ID: 4BDT) were analyzed via molecular docking. Docking scores and Root mean square deviation (RMSD) values predict the strong binding of compounds with AChE. Apigenin‐7‐O‐glucoside depicted minimal docking score as compared to standard, donepezil, hence revealing its strong interaction with enzyme (Figure 14b). Multiple strong hydrogen bonds interactions of Apigenin‐7‐O‐glucoside were analyzed with different amino acid residues (GLY122; ASN87; THR83; GLY120; TYR:133; GLH202) (Figure 14b).Further, the compounds were analyzed to check their physicochemical and chemical absorption, distribution, metabolism, excretion and toxicity (ADMET) properties. The physicochemical characteristics were estimated using the QikProp program. Every compound complied with the Lipinski rule. The estimated values for the octanol/water partition coefficient “QPlogPo/w” range from −0.285 to 8.63. The IC_50_ values for blocking HERG K+ channels “QPlogHERG” vary from −6.757 to −1.432. In contrast, the IC_50_ values for caco‐2 cell permeability “QPPCaco” range from 2.228 to 9906.038, values of brain/blood partition coefficient “QPlogBB” range from 4.622 to 0.675, and human serum albumin binding “QPkhsa” values ranging from −1.18 to 2.254 are within the permitted ranges for 95% of oral drugs. Physical and chemical properties like “QPlogPo/w and QPlogHERG” revealed uniform drug diffusion and defense to acceptable range  . The docking score, physicochemical and ADMET properties of ligands are shown in Tables 4 and 5.

**TABLE 4:** Physicochemical parameters of compounds present in *Morchella esculenta* fruiting bodies and mycelia.

Compounds	MW	HBD	HBA	QPlogPo/w	QPlogHERG	QPPCaco	QPlogBB	QPlogKhsa
Protocatechuic acid	154.122	3	3	0.03	−1.522	26.561	−1.238	−0.897
p‐Hydroxybenzoic acid	138.123	2	2	0.585	−1.633	73.814	−0.804	−0.796
Gallic acid	170.121	4	4	−0.57	−1.432	9.636	−1.681	−0.981
Donepezil	379.498	0	5	4.527	−6.757*	1151.627	0.233	0.622
Ecgonine methyl ester	199.249	0	4	0.507	−3.889	399.721	0.154	−0.675
Atractylol	216.322	0	1	3.581	−3.211	9906.038	0.675	0.741
Apigenin‐7‐O‐glucoside	432.383	5	12	−0.285	−5.969	10.624	−3.22	−0.689
Rhoifolin	578.526	7	19	−1.497	−6.582	2.228	−4.622	−1.18
α‐Tocopherols	416.686	1	1	8.63	−5.892	3675.728	−0.763	2.243
γ‐Tocopherols	416.686	1	1	8.652	−5.815	3676.077	−0.756	2.254
δ‐Tocopherols	416.686	1	1	8.644	−5.863	3676.077	−0.761	2.25

* Molecular weight (MW), Hydrogen bond donor (HBD), Hydrogen bond acceptor (HBA)

**TABLE 5:** Glide score and (Root mean square deviation) RMSD of compounds present in *Morchella esculenta* fruiting bodies and mycelia.

Compounds	Glide score	RMSD
Apigenin‐7‐O‐glucoside	−8.838	3.622
Donepezil	−8.5	3.772
α‐Tocopherols	−7.937	2.689
Atractylol	−7.627	3.996
Protocatechuic acid	−7.61	4.181
Rhoifolin	−7.568	3.503
Gallic Acid	−7.188	3.665
p‐Hydroxybenzoic acid	−6.556	0.129
γ‐Tocopherols	−6.195	4.388
δ‐Tocopherols	−6.195	4.388
Ecgonine methyl ester	−5.631	4.525

## 4 DISCUSSION

This study focused on the potential use of *M. esculenta* extract as an alternative or complementary treatment for various clinical conditions, including neurogenerative disorders. The methanolic extracts (KMFB and KMM) exhibited the highest yield compared to the other extracts. Phytochemicals analysis confirmed the presence of flavonoids, phenols, saponins, alkaloids, and glycosides in all four extracts of *M. esculenta* (Table 2). These findings are consistent with previous studies conducted on *Morchella* species showing the presence of these secondary metabolites in different mushrooms (Cheng et al., 2024) (Dissanayake et al., 2021). The KMFB extract showed the highest TPC (50.63 ± 1.23 mg GAE/100g DW), followed by KMM (42.45 ± 0.73 mg GAE/100g DW), and further stalked by TFB/TM, SKFB/SKM, and MJFB/MJM extracts (Table 3). The TPC values obtained in this study align with previous reports on *Morchella* species, which are rich in phenolic compounds with potential antioxidant and anti‐inflammatory activities (Taşkın et al., 2021). Gursoy et al. (2009), reported that several *Morchella* species (*Morchella rotunda*, *Morchella crassipes*, *Morchella esculenta*, *Morchella deliciosa*, *Morchella elata*, and *Morchella angusticeps)* exhibited the higher concentration of bioactive compounds.

All samples exhibited 65%–90% scavenging activity at a 50 µg/mL concentration. These findings are consistent with previous research that reported the antioxidant activity of phenolic compounds in *Morchella paragenesis* (Mustafa et al., 2019). In the DPPH assay, the fruiting body extract of *M. esculenta* showed higher scavenging activity than the mycelia extract and gallic acid, which is in line with the previous study that reported higher antioxidant activity of the *Morchella* fruiting body (Y. Li et al., 2023). The inclusion of phenolic compounds may be the cause of *M. esculenta*’s increased scavenging activity (Taşkın et al., 2021). Another study reported that phenolic compounds extracted from *Morchella rufobrunnea* can scavenge free radicals (Dissanayake et al., 2021). Sunil et al. (2022) revealed that methanolic extract of *M. esculenta* exhibited potent DPPH radical scavenging activity.

In the ABTS assay, extracts of *M. esculenta* (MJFB/MJM) revealed higher scavenging activity at 100 µg/mL concentration in comparison to gallic acid (Figure 3). Kumla et al. (2021) reported that the ethanolic extract of *Morchella* species had potent antioxidant activity determined by the ferric reducing antioxidant power (FRAP) and 2,2‐diphenyl‐1‐picrylhydrazyl (DPPH) assays. These findings suggest that *M. esculenta* may be helpful in antioxidant therapy and therapeutic intervention for disorders involving oxidative (Mwangi et al., 2022).

In anti‐inflammatory analysis, the fruiting body extract showed higher BSA protein denatinhibitory potential than the mycelia extract and standard drug aspirin. KMFB and TFB samples showed 95% and 86% inhibition activity among all tested samples. The IC_50_ value (10.94 ± 0.098 µg/mL) of the SKFB sample exhibited the lowest IC_50_ values, indicating the highest inhibitory activity (Figure 5). These findings are in accordance with previous research on the extracts' anti‐inflammatory properties from *Morchella spp*. (X. Zhao et al., 2018). *M. esculenta* extracts contain bioactive substances such as polysaccharides, phenolic compounds, and flavonoids, which may play an anti‐inflammatory role (Cao et al., 2020).

In the protein denaturation assay, all the extracts showed 60%–95% inhibition of protein denaturation, indicating their potential anti‐inflammatory activity compared to aspirin. Similarly, previous studies reported the anti‐inflammatory potential of *Morchella* species (Ying et al., 2023). The membrane stabilization test revealed that all extracts significantly protected RBCs from hemolysis, with the fruiting body extract showing the highest percentage of protection and lowest EC_50_ value. This finding is supported by studies on edible mushrooms, as white oyster mushroom extract demonstrated 43.50% protection at a concentration of 500 µg/mL in the human red blood cell membrane stabilization assay (Figures 7 & 8), indicating its protective effects against hemolysis (Prabu et al., 2015). In another study, methanolic extract of the *Calocybe indica* provided 55.83% membrane stabilization, further supporting the role of edible mushrooms in RBC protection and stabilization (Ganesh et al., 2017). According to Liu et al., *Morchella* polysaccharides reduce inflammation in RAW264.7 by blocking the p38 MAPK signaling pathway and serve as immunological adjuvants by boosting the immune response (W. Li, et al., 2023).

The pathogenesis of neurodegenerative diseases involves several mechanisms, including an altered activity of AChE, leading to inflammation, apoptosis, and oxidative stress (Xiong et al., 2016). In this study, KMFB and KMM extracts showed significantly high anti‐AChE activity (IC_50 _= 6.908 ± 0.171 and 8.903 ± 0.004 µg/mL) as compared to the standard drug, Donepezil (IC_50 _= 21.24 ± 0.137 µg/mL) (Figures 9 & 10). Previous studies have also reported the AChE inhibition potential of different mushroom extracts (Neagu et al., 2018). Polysaccharides of *Morchella* species are known to possess AChE inhibition activity (Badshah et al., 2021). Synthetic AChE inhibitors are commonly used to treat neurodegenerative diseases, and the present study suggests the potential usefulness of *M. esculenta* extracts (Sunil et al., 2022; Mani et al., 2022). Significantly lower IC_50_ values of the tested samples compared with standard drugs highlight the novelty of this study.

The enzyme kinetics analysis of the *M. esculenta* extracts revealed that KMFB can be a competitive inhibitor of AChE. The calculated value of *K*i for KMFB was 0.7 µM, indicating potent inhibition activity. Furthermore, the *K*m values decreased significantly with increasing extract concentrations, whereas *V*max remained unaffected, indicating that the extract effectively inhibits AChE activity (Figure 11‐13). Recently, Thu et al. (2020) showed that *Morchella* esculenta extract inhibits enzyme activity with an IC_50_ value of 5.5 mg/mL, indicating its potential as a natural inhibitor. Similarly, another study by J. Zhou et al. (2020) reported the inhibitory activity of *Morchella* species. These findings suggest that *M. esculenta* could be a natural source of AchE inhibitors.

*In silico* analysis was also conducted to explore the binding interactions, physicochemical and ADMET properties of constituents of *M. esculenta*. Apigenin‐7‐O‐glucoside was found to exhibit a strong binding interactions with amino acids residues of AChE as compared to standard drug, donepezil (Table 5) (Figure 14b). Further, the investigated compounds were demonstrated to follow Lipinski's rule, indicating their potential as oral drugs. Another critical parameter assessed in this study was the octanol/water partition coefficient (QPlogPo/w). This parameter provides insights into the compound’s ability to dissolve in hydrophilic and hydrophobic environments. The range of values observed (−0.285 to 8.63) suggests that the compounds have diverse solubilities, indicating their potential to interact with various biological systems (Table 4). These findings align with previous studies that have shown a correlation between appropriate solubility and oral bioavailability of drugs (Bremmell et al., 2019). Another parameter analyzed in this study was the HERG K+ channels blocking IC_50_ (QPlogHERG). The observed range of values (−6.757 to −1.432) suggests that the compounds exhibit different blocking activity on HERG potassium channels. This parameter is particularly relevant to assessing potential cardiotoxicity, as drugs that block HERG channels can lead to cardiac arrhythmias (Garrido et al., 2020). Further investigations are required to determine the specific implications of these results and assess the compounds' cardiotoxic potential.

The caco‐2 cell permeability (QPPCaco) values were also evaluated. Caco‐2 cells are generally utilized as an in vitro model to assess the absorption of compounds in the gastrointestinal tract. The wide range of QPPCaco values (2.228–9906.038) suggests that the compounds exhibit varying levels of permeability. Favorable permeability is critical for achieving optimal oral bioavailability (Lanevskij et al., 2019). The brain/blood partition coefficient (QPlogBB) values provide insights into a compound's ability to cross the blood‐brain barrier. The observed range of values (−4.622 to 0.675) suggests that the compounds possess different capacities to penetrate the central nervous system. This parameter is particularly interesting for drugs targeting neurological disorders, as effective penetration of the blood‐brain barrier is crucial for therapeutic efficacy (Pardridge et al., 2020).

These findings, detailed in Tables 4 and 5 and illustrated in Figures 14a and 14b, underscore the diverse pharmacological profiles of *M. esculenta* compounds. The molecular docking results (Table 4) and the interaction diagrams (Figure 14a,b) highlight specific compounds with strong binding affinities to targeted receptors, reinforcing their potential as drug candidates. These docking results serve as a starting point for experimental validation, as they suggest that compounds such as apigenin‐7‐O‐glucoside, gallic acid, and protocatechuic acid may have therapeutic applications in treating neurodegenerative diseases and cardiovascular conditions. However, further in vitro and in vivo testing is required to confirm their biological activity and safety.

Finally, the human serum albumin binding (QPkhsa) values were assessed. Human serum albumin plays several critical functions in maintaining homeostasis, including regulating drug distribution and elimination. The range of values (−1.18 to 2.254) indicates that the compounds exhibit different degrees of binding affinity to human serum albumin (Table 4). Previous studies have highlighted the significance of protein binding in drug pharmacokinetics and the need to consider the extent of binding during drug development (Y. Zhou et al., 2022). Combined, the bioactive constituents could offer a strong potency for slowing the neurodegenerative progression and may pose conginitive functionality.

## 5 CONCLUSION

The methanolic extracts of *M. esculenta* were found to contain potent phytoconstituents with antioxidant, anti‐inflammatory, and anti‐acetylcholinesterase effects. *In silico* and enzyme kinetics analysis highlighted the AChE inhibition potential and pharmacological behavior of *M. esculenta* extracts. These promising findings support further *in vivo* analysis to explore the mechanistic therapeutic potential of *M. esculenta* and its phytoconstituents.

## AUTHOR CONTRIBUTIONS

**Rida Haider**: Conceptualization; writing—original draft; data curation. **Luisa Agnello**: Writing—review and editing; supervision. **Shahid Masood Shah**: Data curation; formal analysis; writing—original draft. **Muhammad Sufyan**: Data curation; formal analysis; writing—original draft. **Nimra Khan**: Data curation; visualization; formal analysis. **Abdul Nazir**: Data curation; formal analysis; Writing—original draft. **Marcello Ciaccio**: Supervision; writing—review and editing; validation; visualization. **Sidra Rehman**: Writing—review and editing; conceptualization; validation; Supervision.

## CONFLICT OF INTEREST STATEMENT

The authors declare no conflicts of interest.

## Data Availability

Derived data supporting the findings of this study are available from the corresponding author on request.
